# TRIM46 aggravated high glucose-induced hyper permeability and inflammatory response in human retinal capillary endothelial cells by promoting IκBα ubiquitination

**DOI:** 10.1186/s40662-022-00305-2

**Published:** 2022-09-05

**Authors:** Hangqi Shen, Qiaoyun Gong, Jingting Zhang, Haiyan Wang, Qinghua Qiu, Jingfa Zhang, Dawei Luo

**Affiliations:** Department of Ophthalmology, Shanghai General Hospital (Shanghai First People’s Hospital), Shanghai Jiao Tong University School of Medicine, 100 Haining Road, Hongkou District, Shanghai, 200080 China

**Keywords:** Diabetic retinopathy, TRIM46, IκBα, Ubiquitination, NF-κB

## Abstract

**Background:**

Diabetic retinopathy (DR) as a severe diabetic complication contributes to blindness. The increased permeability of retinal capillary endothelial cells (RCECs) as well as the production of inflammatory markers are closely related to DR occurrence. We recently revealed that TRIM46 promotes high glucose (HG)-caused ferroptosis in human RCECs (HRCECs). The current study aims to explore the molecular mechanism of how TRIM46 plays its role in DR progression.

**Methods:**

Western blot was utilized to determine protein expression. The cell counting kit-8 assay was used to observe cell viability. The permeability of the cell layer was determined by measuring the transepithelial electrical resistance and fluorescein isothiocyanate (FITC)-dextran leak. Enzyme-linked immunosorbent assay was used to quantify the protein level of pro-inflammatory cytokines and co-immunoprecipitation was employed to verify the relationship between TRIM46 and IκBα.

**Results:**

HG dramatically upregulated TRIM46 protein expression in a dose-dependent way. Silencing TRIM46 effectively reversed HG-induced cell growth inhibition, cell cycle arrest, hyper permeability and pro-inflammatory cytokines secretion in HRCECs, while overexpression of TRIM46 exhibited an opposite effect. Furthermore, TRIM46 was able to interact with IκBα and promote the ubiquitination and degradation of IκBα. IκBα overexpression recovered the effects of TRIM46 overexpression in HRCECs. Furthermore, inhibiting the activation of NF-κB partially recovered HG-induced HRCEC injury, whereas TRIM46 overexpression reversed these effects.

**Conclusion:**

This study demonstrates that TRIM46 interacts with IκBα to activate the NF-κB signaling pathway, thereby enhancing cell proliferation inhibition, hyper permeability and the inflammatory response of HRCECs in a HG state.

**Supplementary Information:**

The online version contains supplementary material available at 10.1186/s40662-022-00305-2.

## Background

Diabetes as a common chronic disease causes blood glucose levels to rise. The International Diabetes Federation (IDF) revealed that the population of sufferers diagnosed with diabetics will elevate by 54.7% by 2040 [1]. Persistent hyperglycemia contributes to systemic microvascular disease, thereby leading to chronic complications of diabetes including neuropathy, kidney disease and diabetic retinopathy (DR) [2, 3]. DR is identified as the leading cause of blindness in the middle-aged and the elderly, and is a hallmark of microvascular complications associated with diabetes [4, 5]. Macular edema in non-proliferative DR is one of the vital causes of early vision loss. Once proliferative DR develops, visual function is often severely impaired. Although anti-neovascularization drugs, laser therapy and surgical treatment reduce morbidity, DR still seriously impacts vision and patients’ quality of life. Patients with DR typically have a low cure rate, high rate of blindness and poor postoperative visual function recovery [6, 7]. Therefore, it is critical to uncover the complicated pathogenesis of DR to prevent and treat DR.

The blood-retinal barrier (BRB) protects the retina by removing toxic substances and nerve components. High glucose (HG) stimulation contributes to a variety of pathologies in the retina such as oxidative stress, inflammatory response and damage to the BRB, which allow lipids and fluids to penetrate the retina and aggravate DR [8–11].

Nuclear factor kappa B (NF-κB) is a vital transcription factor involved in a diverse range of cellular activities including inflammation and carcinogenesis [12]. When the NF-κB signaling pathway is stimulated, inhibitory κB (IκB) kinase (IKK) is activated. The activated IKK is able to phosphorylate IκB, leading to ubiquitination and degradation of IκBα [13]. NF-κB is then released from IκB in the cytoplasm, and further translocate into the nucleus to activate transcription of downstream genes including inflammatory markers, tumor necrosis factor alpha (TNF-α), interleukin (IL)-1β as well as IL-6 [13–15]. Previous studies indicated that the NF-κB pathway is tightly interlinked with the progression of DR [16, 17], however, the proteins associated with the ubiquitination of IκBα in DR pathogenesis has not been uncovered.

The E3-ligase tripartite motif (TRIM) family exerts vital effects on multiple biological activities. For example, it was reported that TRIM52 regulates NF-κB signaling pathway [18]. TRIM67 inhibited colorectal cancer development by mediating tumor protein p53 [19]. Besides, TRIM46 as a member of TRIM family is associated with various cellular functions such as cancer cell proliferation, cell cycle and serum uric acid levels [20–22]. In addition, it was reported that TRIM46 facilitates growth and reduces the rate of apoptosis in osteosarcoma cells by modulating the NF-κB pathway [21]. Our recently published study also revealed that TRIM46 promotes HG-caused ferroptosis and cell proliferation suppression in human retinal capillary endothelial cells (HRCECs) through the acceleration of glutathione peroxidase 4 (GPX4) degradation [7]. However, whether and how TRIM46 affects the BRB, inflammatory response and NF-κB signaling pathway is unclear.

Here, we explored the role of TRIM46 in DR progression. The data illustrates that TRIM46 accelerated the ubiquitination of IκBα and activated the NF-κB signaling pathway, which further inhibited the cell viability and upregulated permeability and inflammation response of HRCECs in a high glucose environment. These findings also provide a fundamental basis for TRIM46 as a therapeutic target in the treatment of DR.

## Materials and methods

### Cell culture

HRCECs bought from the Type Culture Collection of the Chinese Academy of Science were maintained in Dulbecco's Modified Eagle Medium (10566024, Thermo Scientific, USA) containing 10% fetal bovine serum (10099141, Thermo Scientific, USA) in a 37 °C culture incubator supplemented with 5% CO_2_.

### High glucose-induced model

HRCECs (2 × 10^5^/well) were plated in 6-well plates. The cells were then treated with 10, 15 or 25 mM glucose for 24 h. The control cells were treated using 5.5 mM glucose and mannitol (M2069, Sigma Aldrich, USA) to control osmotic pressure.

### Quantitative real-time PCR (qRT-PCR)

TRIzol (10,296,010, Thermo Scientific, USA) was used to isolate the total RNA in HECECs following the manufacturer’s protocol. The qRT-PCR analysis was conducted using SYBR^®^Green PCR Master Mix (4309155, Thermo Scientific, USA) on the ABI 7300 instrument (Applied Biosystems) and β-actin was used as the reference gene. The relative expression level of a specific gene was calculated by employing 2^−ΔΔCt^ method. The primers used are listed here:

TRIM46-Forward-5′-CTGCTTGAGAACCCCGACA-3′,

TRIM46-Reverse-5′GCTCGCTGGTCCTTGCTG-3′;

IκBα-Forward-5′-CACCAACCAGCCAGA AAT-3′,

IκBα-Reverse-5′-ACCCAAGGACACCAAAAG-3′;

β-actin-Forward-5′-TGGCATTGCCGACAGG-3′,

β-actin-Reverse-5′-GCATTTGCGGTGGACG-3′.

### Western blot

Total protein was harvested using the radio-immunoprecipitation assay (RIPA) lysis and extraction buffer (89901, Thermo Scientific, USA) containing proteinase inhibitor cocktail (87786, Thermo Scientific, USA). The NE-PER™ Nuclear and Cytoplasmic Extraction Reagents (78835, Thermo Fisher Scientific, USA) was then used to isolate cytoplasmic and nuclear proteins following manufacturer’s protocol. Proteins were separated using sodium dodecyl sulfate–polyacrylamide gel electrophoresis and transferred into polyvinylidene fluoride membrane (PVDF membrane, 88518, Thermo Fisher Scientific, USA). After blocking with 5% skim milk in phosphate buffer saline (PBS), specific primary antibodies were used to incubate with above PVDF membrane at 4 °C. After incubation overnight, the membrane was washed for 3 times using PBS and was incubated with appropriate Horseradish peroxidase (HRP)-conjugated secondary antibodies (A0208, Beyotime, China) for 2 h. The protein bands were detected using HRP chemiluminescence substrate (A38555, Thermo Scientific, USA) with the iBright imaging system (iBright CL1500, Invitrogen, USA). The primary antibodies are as follows: TRIM46 (21026-1-AP, Proteintech, USA, 1:1000), IκBα (Ab76429, Abcam, UK, 1:1000), zona occludens 1 (ZO-1) (Ab96587, Abcam, UK, 1:1000), Occludin (Ab167161, Abcam, UK, 1:1000), NF-κB (Ab16502, Abcam, UK, 1:1000), H3 (17168-1-AP, Proteintech, USA, 1:1000), and β-actin (66009-1-lg, Proteintech, USA, 1:1000).

### Short hairpin RNA (shRNA)-mediated interference

ShRNA oligonucleotides paired with TRIM46 were cloned into the pLKO.1 plasmid (10878, Addgene, USA). The successful construction of plasmids was verified via DNA sequencing. The targeted sequences are as listed here:

shTRIM46-1-GGAGAGCAAGCUUCAAGAATT;

shTRIM46-2-CAUGGUUUAUAAACAAUAATT;

shTRIM46-3-GGGCUGUGCUGGAGGAGAATT.

Then, 293 T cells were transfected using the above plasmids containing shTRIM46-1, shTRIM46-2, or shTRIM46-3, and the packaging plasmids psPAX2 and pMD2G by employing Lipofectamine 2000 (11668019, Invitrogen, USA). After incubation for 48–72 h, the generated lentiviruses were harvested for subsequent assays.

### Overexpression of TRIM46 and IκBα

The complementary DNA (cDNA) of TRIM46 or IκBα was constructed into pcDNA3.1 vector (Life Technologies). The successful construction of plasmids was verified via DNA sequencing. Then, HRCECs were transfected with the plasmids using Lipofectamine 2000 (11668019, Invitrogen, USA).

### Cell counting kit-8 (CCK-8) assay

Briefly, 10 µL CCK-8 solution (96992, Sigma-Aldrich, USA) was utilized to incubate with HRCECs as described in the manufacturer’s protocol for 4 h. Finally, a Multiskan™ FC microplate tester (51119180ET, Thermo Scientific, USA) was used to read the absorbance at 450 nm.

### Cell cycle analysis

HRCECs treated as indicated were collected and fixed with ice-cold ethanol at 4 °C overnight. After labelling with propidium iodine (PI, Sigma-Aldrich), cell cycle was analyzed with flow cytometry (BD Biosciences, Franklin Lakes, NJ, USA) following the manufacturer’s instructions.

### Determination of TNF-α, IL-6 and IL-1β

The protein levels of TNF-α, IL-6 and IL-1β secreted via HRCECs was measured using specific enzyme-linked immunosorbent assay (ELISA) kits (BMS223HS, BMS213-2 and BMS224-2, Invitrogen, USA) according to the manufacturer's protocol.

### Transepithelial electrical resistance (TEER) assay and fluorescein isothiocyanate (FITC)-dextran assay

HRCECs (1 × 10^4^ cells/well) were cultured in Transwell filters (pore size, 0.4 µm, CLS3396, Corning, USA) in a cell incubator at 37 °C supplemented with 5% CO_2_. After the cells grew to confluence, and were treated as indicated, TEER was determined utilizing the Millicell-ERS2 Volt-Ohm Meter (Millipore, USA) according to the manufacturer’s instructions. The TEER value (Ω cm^2^) was obtained by removing the resistance of the base filter and correcting for the surface area (0.6 cm^2^). Normalized TEER was determined as the ratio of the treated group’s TEER to the control group’s TEER.

After the cells grew to confluence, the cells were treated as indicated for 0 and 24 h. FITC-conjugated dextran (1 μg/μL, MW 70 000; 53471, Sigma-Aldrich, USA) was added to the upper chamber for 2 h. Then, a 100 μL mixture in the lower chamber was harvested and were detected using a spectrofluorometer (970CRT, YiTian, China). A permeability coefficient (PC) for FITC-dextran was determined as follows: PC (cm/min) = V/(SA × C0) × (Ct/T) [23], where V is the volume of medium in the lower chamber, SA is the surface area (0.6 cm^2^), C0 is the concentration of FITC-conjugated dextran in the upper chamber at time 0, and Ct is the concentration of FITC-conjugated dextran in the lower chamber at sampling time T.

### Co-immunoprecipitation (Co-IP)

Cell lysates were incubated with antibodies TRIM46 (21026-1-AP, Proteintech, USA, 1:500), IκBα (Ab76429, Abcam, UK, 1:500) or IgG (sc-69786, Santa Cruz Biotech, USA, 1:500) for 1 h. The mixture was then incubated with Pierce Protein A/G Plus Agarose (20423, Thermo Scientific, USA) for another 3 h. All incubation processes were carried out at 4 °C. Finally, the precipitate was washed using washing buffer for three times and then analyzed via Western blotting.

### Dual-luciferase reporter gene assay

The sequence of the TRIM46 promoter was cloned into a pGL3 vector (Promega, USA). HRCECs were transfected with the pGL3-TRIN46 promoter and pRL-SV40 vector using Lipofectamine 2000, and then exposed to high glucose (HG, 25 mM) and 10 μM NF-κB inhibitor pyrrolidine dithiocarbamate (PDTC) (HY-18738, MedChemExpress, USA) for 24 h. Luciferase activity was detected using the Luciferase Reporter Gene Detection Kit (LUC1, Sigma-Aldrich, USA) and a Multiskan™ FC microplate tester (51119180ET, Thermo Scientific, USA).

### Statistical analysis

Statistical data analysis was performed using GraphPad Prism 8 (GraphPad, USA). All data are presented in mean ± standard deviation (SD) and were compared utilizing one-way ANOVA followed by Tukey’s post hoc test. ^*^P < 0.05, ^**^P < 0.01 and ^***^P < 0.001 represent statistically significant differences. All experiments were conducted in at 3 biological replicates.

## Results

### TRIM46 enhanced HG-induced cell growth inhibition, hyper permeability and secretion of pro-inflammatory factors in HRCECs

**Fig. 1:**
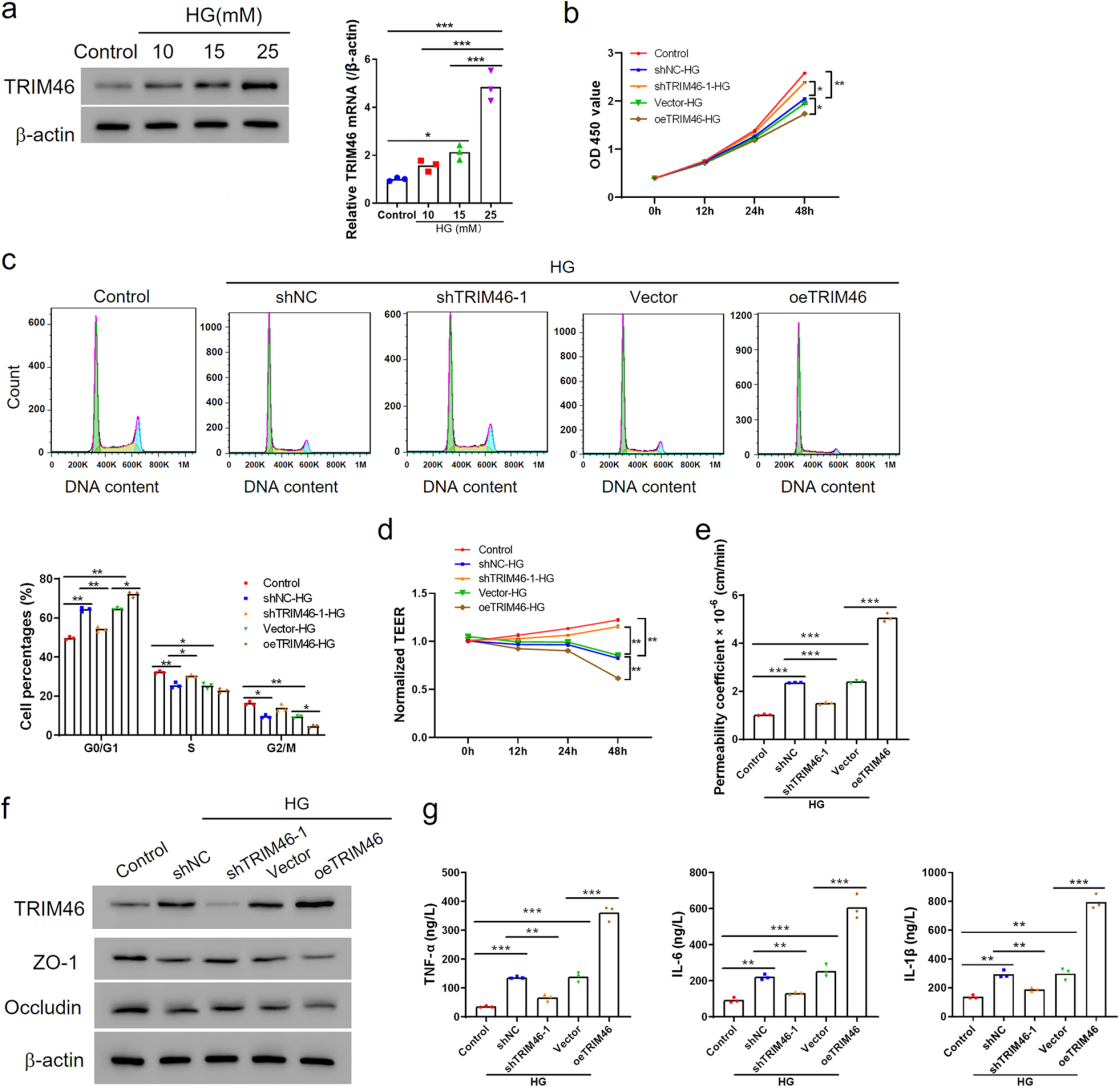
TRIM46 enhanced HG-induced cell growth inhibition, hyper permeability and pro-inflammatory factors secretion in HRCECs. **a** 100% confluent HRCECs were treated with different concentrations of glucose (10, 15, 25 mM). Normal glucose concentration (5.5 mM) and mannitol were used to control the osmotic pressure of control cells. Western blot and qRT-PCR were used to detect the expression of TRIM46 after 24 h of treatment. **b**–**f** In HRCECs, TRIM46 was interfered with or overexpressed for 24 h, and then treated with HG (25 mM). Control osmotic pressure was controlled with normal glucose concentration of 5.5 mM and mannitol. Proliferation and cell cycle was detected with the CCK-8 assay (**b**) and flow cytometry (**c**), respectively. Cell permeability was analyzed by transmembrane electrical resistance (TEER) (**d**) and FITC-dextran leak assay were performed after the cells grew to confluence (**e**). **f** Western blot show TRIM46, Occludin and ZO-1 expression levels. **g** The levels of TNF-α, IL-1β and IL-6 in the supernatant was determined by ELISA. ^*^P < 0.05, ^**^P < 0.01, ^***^P < 0.001. CCK, cell counting kit; ELISA, enzyme-linked immunosorbent assay; FITC, fluorescein isothiocyanate; HG, high glucose; HRCECs, human retinal capillary endothelial cells; qRT-PCR, quantitative real-time PCR; TRIM, tripartite motif

A HG-induced cell injury model in HRCECs was used; different concentrations of glucose were employed as described in our previous study [7]. qRT-PCR and Western blot analysis showed that HG treatment increased the mRNA and protein levels of TRIM46 in a dose-dependent manner, especially at a concentration of 25 mM (Fig. 1a). To explore the role of TRIM46 in HG-induced cell proliferation, permeability and inflammatory response in HRCECs, we transfected HRCECs with a vector overexpressing TRIM46 (oeTRIM46) and determined the successful overexpression efficiency compared with a blank vector (Additional file 1: Fig. S1a). Additionally, we introduced a lentivirus carrying with shTRIM46-1, shTRIM46-2 or shTRIM46-3 to reduce TRIM46 expression. As expected, all three shRNAs decreased TRIM46 protein levels, especially shTRIM46-1 (Additional file 1: Fig. S1b). Therefore, we employed HRCEC-shTRIM46-1 cell lines for the following experiments.

We next explored whether TRIM46 affected HRCECs growth. HG combined with the control vector dramatically inhibited cell proliferation and induced G1 arrest. Overexpression of TRIM46 further inhibited cell proliferation and cell cycle progression, while silencing of TRIM46 recovered cell proliferation remarkably (Fig. 1b, c). Moreover, HG significantly lower TEER at 12 h, 24 h and 48 h, especially at 48 h compared with 0 h. Meanwhile, FITC-dextran leak was upregulated at 24 h by HG treatment compared to basal conditions. TRIM46 overexpression further decreased TEER, whereas TRIM46 knockdown effectively eliminated the reduction of TEER at different times. Besides, silencing of TRIM46 reversed the increase of FITC-dextran leak induced by HG but overexpression TRIM46 aggravated it at 24 h remarkably (Fig. 1d, e; Additional file 1: Fig. S2a). Next, the levels of tight junction proteins (TJPs), ZO-1 and Occludin, were measured to determine the integrity of the BRB. As indicated by Western blot and immunofluorescence staining, HG significantly decreased the protein levels of ZO-1 and Occludin. Overexpression of TRIM46 further downregulated their expression levels, while TRIM46 inhibition elevated ZO-1 and Occludin expression (Fig. 1f; Additional file 1: Fig. S3a). These results suggest that TRIM46 impaired the stability of cell–cell junctions and contributes to the hyper permeability of HRCECs.

Subsequently, to investigate the effects of TRIM46 on inflammation, we quantified the levels of pro-inflammatory cytokines released including TNF-α, IL-6 and IL-1β. The data showed that TRIM46 knockdown recovered the production of pro-inflammatory induced by HG treatment, whereas TRIM46 overexpression enhanced it significantly (Fig. 1g). Taken together, all these findings suggested that TRIM46 enhanced HG-induced cell growth inhibition, and induced hyper permeability and the production of pro-inflammatory factors.

### TRIM46 interacted with IκBα and promotes IκBα ubiquitination in HRCECs

**Fig. 2:**
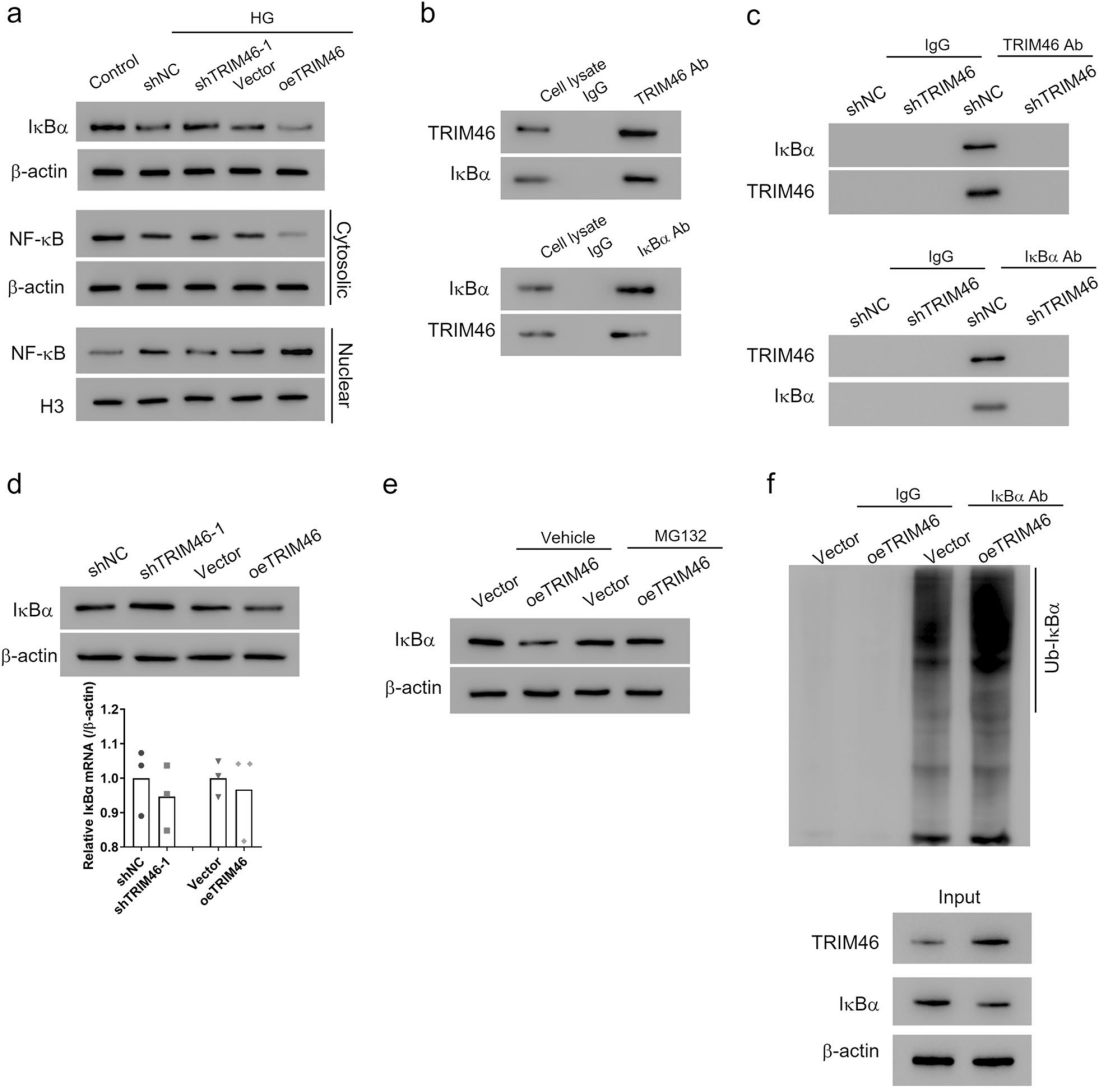
TRIM46 interacts with IκBα and promotes IκBα ubiquitination in HRCECs. **a** Interference or overexpression of TRIM46 for 24 h was followed by treatment with HG (25 mM). Control osmotic pressure was controlled with a normal glucose concentration of 5.5 mM and mannitol. Western blot detected the protein level of NF-κB in the nucleus and plasma and of IκBα. **b**, **c** The interaction between TRIM46 and IκBα detected by Co-IP assay. **d** After interference or overexpression of TRIM46 in HRCECs, the protein and mRNA levels of IκBα were detected by Western and qRT-PCR. **e** HRCECs were transfected with oeTRIM46 or Vector and the expression of IκBα was detected by Western blot after treatment with 10 μmol/L MG132 or Vehicle (DMSO) for 4 h. **f** The ubiquitination of IκBα was detected by IP assay. CCK, cell counting kit; HG, high glucose; HRCECs, human retinal capillary endothelial cells; NF-κB, nuclear factor kappa B; qRT-PCR, quantitative real-time PCR; TRIM, tripartite motif

Given that the NF-κB signaling pathway exerts vital roles in DR and the inflammatory response, we assumed that TRIM46 is the mediator of the NF-κB signaling pathway. To verify the hypothesis, we detected whether TRIM46 affected the protein levels of IκBα and NF-κB. As shown in Fig. 2a, TRIM46 overexpression further reduced the protein levels of IκBα in a HG state, while TRIM46 silence restored IκBα expression. Meanwhile, HG treatment downregulated the protein expression of NF-κB in the cytoplasm, but significantly increased its expression in the nucleus. Overexpression of TRIM46 enhanced these effects while knockdown of TRIM46 notably reversed them (Fig. 2a). These findings indicated that TRIM46 overexpression could further enhance the activated NF-κB pathway induced by HG treatment but silencing of TRIM46 inhibited the activation of this pathway. As the essential inhibitor of NF-κB, we wondered whether TRIM46 regulated IκBα directly. The results demonstrated that TRIM46 was able to co-precipitate with IκBα and vice versa (Fig. 2b). When TRIM46 expression was knocked down, TRIM46 antibody could not precipitate IκBα and vice versa (Fig. 2c). To elucidate how TRIM46 influenced IκBα expression, we measured the protein and mRNA levels of IκBα. Overexpression of TRIM46 reduced the protein expression of IκBα protein level while silencing TRIM46 had the opposite effect. However, the consistent results were not observed at the mRNA level (Fig. 2d). Moreover, after cells were treated with MG132, a commonly used proteasome inhibitor, TRIM46 overexpression could not decrease IκBα expression (Fig. 2e). TRIM46 was also shown to significantly increase IκBα ubiquitination (Fig. 2f). These findings suggested that TRIM46 promoted IκBα ubiquitination and proteasome-dependent degradation.

### IκBα overexpression reversed the effects of TRIM46 on cell growth inhibition, enhancement of hyper permeability and inflammatory response in HRCECs

**Fig. 3:**
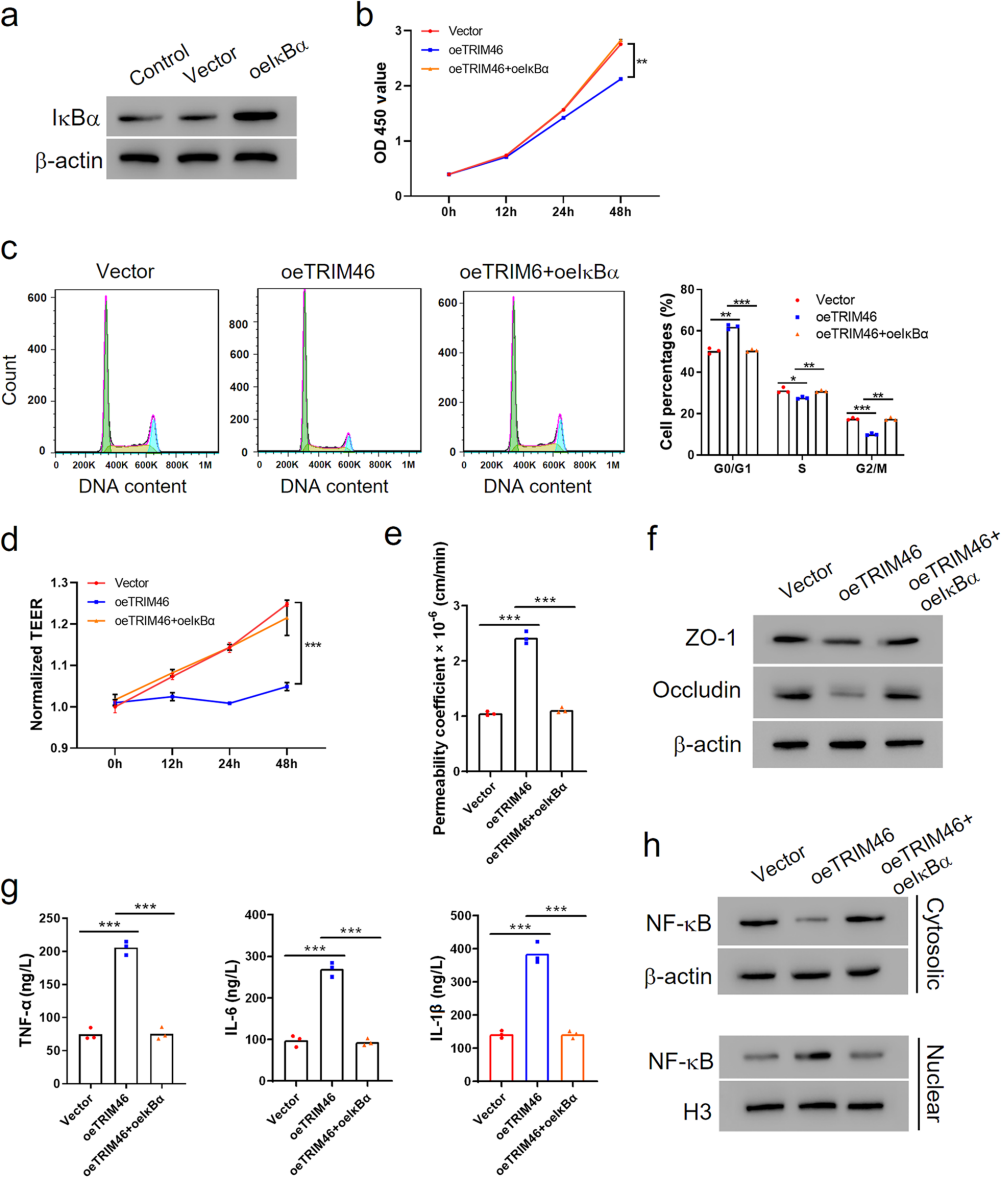
IκBα overexpression reversed the effects of TRIM46 on cell growth inhibition, hyper permeability and inflammatory response in HRCECs. **a** Protein level of IκBα in HRCECs transfected with oeTRIM46 or Vector. **b**–**g** HRCECs transfected with oeTRIM46 or Vector with overexpression of IκBα (oeIκBα). Cell proliferation and cell cycle was detected with CCK-8 assay (**b**) and flow cytometry (**c**), respectively. **d** Transmembrane electrical resistance and (**e**) FITC-dextran leak assay was conducted after the cells grew to confluence. **f** Western blot was used to detect the protein levels of Occludin and ZO-1. **g** The levels of TNF-α, IL-1β and IL-6 in supernatant were determined by ELISA. **h** Western blot was used to detect the protein level of NF-κB in the nucleus and plasma and of IκBα. ^*^P < 0.05, ^**^P < 0.01, ^***^P < 0.001. CCK, cell counting kit; ELISA, enzyme-linked immunosorbent assay; FITC, fluorescein isothiocyanate; HRCECs, human retinal capillary endothelial cells; NF-κB, nuclear factor kappa B; TRIM, tripartite motif

To verify whether TRIM46 inhibited cell proliferation and increased permeability and the inflammatory response in HRCECs by facilitating IκBα degradation, we transfected HRCECs using a vector expressing IκBα and confirmed its transfection efficiency (Fig. 3a). Subsequent assays suggested that overexpression of IκBα recovered the effects of TRIM46 overexpression on cell viability, cell cycle progression, TEER, FITC-dextran leak, and ZO-1 and Occludin expression levels remarkably (Fig. 3b–f; Additional file 1: Figs. S2b, S3a). Additionally, IκBα overexpression effectively reduced the amount of increased inflammatory cytokines, and increased NF-κB nuclear translocation activated by the overexpression of TRIM46 (Fig. 3g, h). Collectively, these data indicate that TRIM46 regulated proliferation, permeability and the inflammatory response of HRCECs at least partly by promoting the degradation of IκBα.

### NF-κB mediated TRIM46 expression and was involved in the effects of TRIM46 on HRCECs

**Fig. 4:**
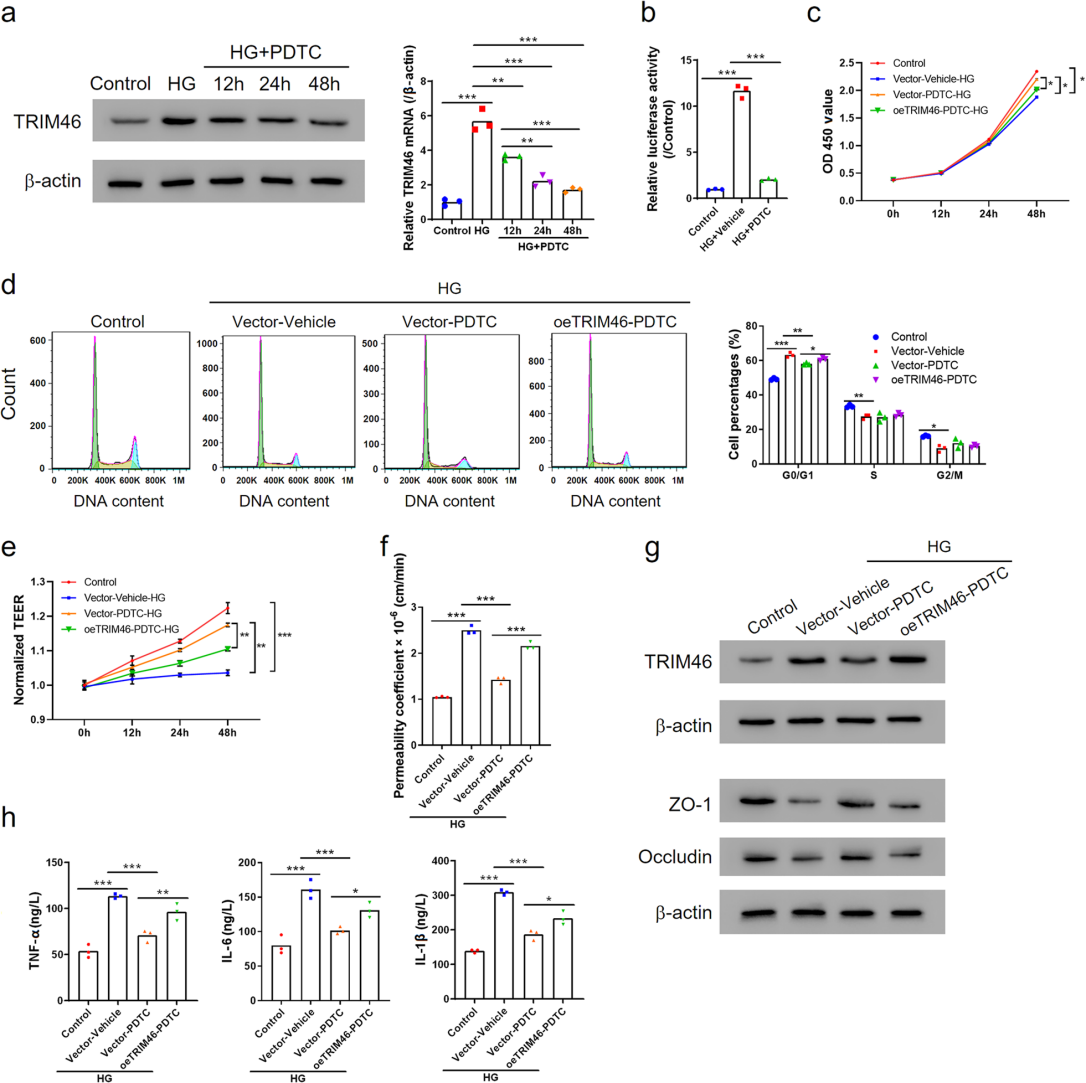
NF-κB mediated TRIM46 expression and is involved in the effects of TRIM46 on HRCECs. **a** TRIM46 expression in HRCECs was detected by Western blot and qRT-PCR after treatment with HG (25 mM) and 10 μM NF-κB inhibitor PDTC at different time points. **b** HRCECs were treated with HG (25 mM) and treated with 10 μM NF-κB inhibitor PDTC for 24 h. Transcriptional activity of TRIM46 promoter was analyzed by the luciferase reporter gene assay. **c**–**h** HRCECs were transfected with oeTRIM46 or Vector and were administered with NF-κB inhibitor PDTC (10 μM) and HG (25 mM). Cell proliferation and cell cycle was detected with CCK-8 assay (**c**) and flow cytometry (**d**), respectively. Transmembrane electrical resistance assay (**e**) and FITC-dextran leak assay (**f**) for analyzing cell permeability were performed after the cells grew to confluence. **g** Western blot showing the expression levels of TRIM46, Occludin and ZO-1. **h** ELISA used for the detection of TNF-α, IL-1β and IL-6 levels in supernatant of cells. ^*^P < 0.05, ^**^P < 0.01, ^***^P < 0.001. HG, high glucose; CCK, cell counting kit; ELISA, enzyme-linked immunosorbent assay; FITC, fluorescein isothiocyanate; HRCECs, human retinal capillary endothelial cells; NF-κB, nuclear factor kappa B; PDTC, pyrrolidine dithiocarbamate; TRIM, tripartite motif

To explore the effects of NF-κB signaling pathway inactivation on the roles of TRIM46 in HRCECs, we introduced PDTC to inhibit the activation of NF-κB. PDTC significantly reduced TRIM46 expression increased by HG treatment at 12 h, 24 h, and 48 h at the protein and mRNA levels especially at 48 h (Fig. 4a). Consistently, luciferase reporter gene analysis illustrated that PTDC suppressed the transcriptional activity of TRIM46 that was significantly upregulated under a HG state (Fig. 4b). Functional analysis showed that PDTC effectively counteracted the roles of HG in cell viability, cell cycle progression, TEER, FITC-dextran leak, and ZO-1 and Occludin expression, whereas TRIM46 overexpression partly reversed these effects (Fig. 4c–g; Additional file 1: Figs. S2c, S3c). Further investigation indicated that PDTC decreased the levels of pro-inflammatory cytokines (TNF-α, IL-6 and IL-1β) secretion and TRIM46 overexpression partially recovered the production of these markers (Fig. 4h). Therefore, these findings suggest that NF-κB inactivation was tightly associated with the roles of TRIM46 in HRCECs.

## Discussion

This investigation revealed for the first time that overexpression of TRIM46, a member of the TRIM family, can further inhibit proliferation and cell cycle progression, as well as increase permeability and the production of pro-inflammatory factors in HRCECs under a HG state. In terms of molecular mechanisms, TRIM46 interacted with IκBα and promoted the ubiquitination and degradation of IκBα. Overexpression of IκBα effectively reversed the effects of TRIM46 overexpression on HRCECs. Additionally, inactivating NF-κB via PDTC also partially offset the effects of HG on HRCECs, which could be reversed by TRIM46 overexpression.

Although controlling blood sugar and blood pressure could alleviate diabetes, vascular-related complications caused by diabetes are still common [24, 25]. DR is identified as the most frequent complication of diabetes with a complex pathogenesis. It was reported that chronic HG might cause BRB injury and DR [26], and thus it is important to confirm the detailed biomarkers during DR progression. Our previous study verified that TRIM46 accelerates HG-caused ferroptosis in HRCECs by regulating ubiquitination and degradation of GPX4 [7]. Consistently, the current study suggested that TRIM46 overexpression aggravated HRCECs proliferation inhibition induced by HG. Increased vascular permeability in the early period of DR leads to later macular edema and is associated with the release of pro-inflammatory cytokines [27]. Besides, Huang et al. found that inflammatory maker TNF-α exerts damaging effects on BRB resolution [28]. Interestingly, our study indicated that overexpression of TRIM46 dramatically upregulated Occludin and ZO-1 expression and increased pro-inflammatory factors production, thereby further enhancing the HG-induced hyper permeability in HRCECs. Contrary results were observed after silencing TRIM46. These results provide a potential molecular basis for TRIM46 to regulate DR processes.

The NF-κB signaling pathway is associated with diverse biological activities including the inflammatory response and DR [29, 30]. IκBα as the strongest inhibitor of NF-κB ensures the activation and closure of the NF-κB pathway [31]. Previous findings have uncovered that some members of the TRIM family like TRIM52 [32] and TRIM38 [33] were involved in regulating the NF-κB signaling pathway. To our knowledge, our study was the first to suggest that TRIM46 overexpression notably reduced the expression of IκBα and promoted the activation of the NF-κB pathway. As a RING finger E3 ligase, TRIM46 possesses the potential to mediate ubiquitin modification by directly binding to substrate proteins [34, 35]. Here, we confirmed that TRIM46 interacted with IκBα and overexpression of TRIM46 facilitated IκBα degradation by increasing its ubiquitination. Further study suggested that IκBα overexpression effectively recovered the effects of TRIM46 overexpression on HRCECs, which confirmed that TRIM46 aggravated the injury of HRCECs in a HG condition by promoting the degradation of IκBα.

Furthermore, NF-κB inactivation effectively decreased HG-induced transcription and expression of TRIM46. Meanwhile, suppressing NF-κB activity also partially recovered HG-induced HRCEC injury. However, NF-κB inhibitors reversed these effects partly by TRIM46 overexpression, which suggested that TRIM46 functions in HRCECs at least in part by modulating the NF-κB signaling pathway. Our current work not only enriched the molecular network of TRIM46 affecting DR process, but also provided substantial theoretical basis for the role of TRIM46 in other cellular activities.

Limitations exist for the current study. First, only in vitro experiments with one cell line were performed to investigate the functions of TRIM46. Whether TRIM46 is able to affect DR process by modulating IκBα and NF-κB pathway in vivo still needs to be explored in the future. Second, both long-term [36] and short-term exposure [7] to HG decreased cell viability in rat retinal endothelial cells. Persistent hyperglycemia is closely related with DR, and thus it is worthwhile to explore the possible functions of TRIM46 during longer-term stimulation with HG. Additionally, how TRIM46 mediates ubiquitination of IκBα needs further study.

## Conclusion

In summary, the current study suggested that TRIM46 promoted the ubiquitination and degradation of IκBα and induced the activation of the NF-κB signaling, thereby exacerbating HG-induced growth inhibition, cytokine storms and hyper permeability in HRCECs. Our study revealed a fundamental molecular mechanism by which TRIM46 regulates the progression of DR, and also provided a potential therapeutic target for the treatment of DR.

## Supplementary Information

**Additional file 1**: **Fig. S1** TRIM46 overexpression and short hairpin RNA (shRNA)-mediated interference of TRIM46 in HRCECs. **a** Protein expression of TRIM46 in HRCECs transfected with oeTRIM46 or Vector. **b** Protein expression of TRIM46 in HRCECs transduced with TRIM46 shRNAs (shTRIM46-1, shTRIM46-2 and shTRIM46-3) or control shRNA (shNC). HRCECs, human retinal capillary endothelial cells; TRIM, tripartite motif. **Fig. S2. **The images of the monolayers are shown (scale bar: 25 μm). **a** In HRCECs, TRIM46 was interfered with or overexpressed for 24 h. After the cells grew to confluence, the cells were then treated with HG (25 mM). Control osmotic pressure was controlled with normal glucose concentration of 5.5 mM and mannitol. **b** HRCECs transfected with oeTRIM46 or Vector with overexpression of IκBα (oeIκBα). After the cells grew to confluence, cell permeability analysis was performed (Figure 3d-e). **c** HRCECs were transfected with oeTRIM46 or Vector. After the cells grew to confluence, cells were administered with the NF-κB inhibitor PDTC (10 μM) and HG (25 mM). Cell permeability analysis was then performed (Figure 4e-f). HG, high glucose; HRCECs, human retinal capillary endothelial cells; NF-κB, nuclear factor kappa B; PDTC, pyrrolidine dithiocarbamate; TRIM, tripartite motif. **Fig. S3. **Immunofluorescence staining of tight junction proteins ZO-1 and Occludin. Blue: DAPI, Green: ZO-1 or Occludin. Scale bar: 50 μm. **a** In HRCECs, TRIM46 was interfered with or overexpressed for 24 h. After the cells grew to confluence, the cells were then treated with HG (25 mM). Control osmotic pressure was controlled with normal glucose concentration of 5.5 mM and mannitol. **b** HRCECs transfected with oeTRIM46 or Vector with overexpression of IκBα (oeIκBα). After the cells grew to confluence, cell permeability analysis was performed (Figure 3d-e). **c** HRCECs were transfected with oeTRIM46 or Vector. After the cells grew to confluence, cells were administered with the NF-κB inhibitor PDTC (10 μM) and HG (25 mM). Cell permeability analysis was then performed (Figure 4e-f). HG, high glucose; HRCECs, human retinal capillary endothelial cells; NF-κB, nuclear factor kappa B; PDTC, pyrrolidine dithiocarbamate; TRIM, tripartite motif.

## Data Availability

All data generated or analyzed during this study are included in this published article and its supplementary information.

## Abbreviations

IDF: International Diabetes Federation
DR: Diabetic retinopathy
BRB: Blood-retinal barrier
HG: High glucose
NF-κB: Nuclear factor kappa B
PDTC: Pyrrolidine dithiocarbamate
GPX4: Glutathione peroxidase
IκB: Inhibitory κB
IKK: IκB kinase
TNF-α: Tumor necrosis factor alpha
IL: Interleukin
TRIM: Tripartite motif
HRCECs: Human retinal capillary endothelial cells
ZO-1: Zona occludens 1
CCK-8: Cell counting kit-8
ELISA: Enzyme-linked immunosorbent assay
qRT-PCR: Quantitative real-time PCR
TEER: Transepithelial electrical resistance
FITC: Fluorescein isothiocyanate
RIPA: Radio-immunoprecipitation assay
IP: Immunoprecipitation
SD: Standard deviation
PBS: Phosphate buffer saline
HRP: Horseradish peroxidase
