# Supplementary material for: TRIM46 aggravated high glucose-induced hyper permeability and inflammatory response in human retinal capillary endothelial cells by promoting IκBα ubiquitination
Source: Eye Vis (Lond). 2022 Sep 5;9:35. doi: 10.1186/s40662-022-00305-2 (PMC9443035; doi:10.1186/s40662-022-00305-2)
Supplement: Supplementary file 1 — Additional file 1: Fig. S1 TRIM46 overexpression and short hairpin RNA (shRNA)-mediated interference of TRIM46 in HRCECs. a Protein expression of TRIM46 in HRCECs transfected with oeTRIM46 or Vector. b Protein expression of TRIM46 in HRCECs transduced with TRIM46 shRNAs (shTRIM46-1, shTRIM46-2 and shTRIM46-3) or control shRNA (shNC). HRCECs, human retinal capillary endothelial cells; TRIM, tripartite motif. Fig. S2. The images of the monolayers are shown (scale bar: 25 μm). a In HRCECs, TRIM46 was interfered with or overexpressed for 24 h. After the cells grew to confluence, the cells were then treated with HG (25 mM). Control osmotic pressure was controlled with normal glucose concentration of 5.5 mM and mannitol. b HRCECs transfected with oeTRIM46 or Vector with overexpression of IκBα (oeIκBα). After the cells grew to confluence, cell permeability analysis was performed (Figure 3d-e). c HRCECs were transfected with oeTRIM46 or Vector. After the cells grew to confluence, cells were administered with the NF-κB inhibitor PDTC (10 μM) and HG (25 mM). Cell permeability analysis was then performed (Figure 4e-f). HG, high glucose; HRCECs, human retinal capillary endothelial cells; NF-κB, nuclear factor kappa B; PDTC, pyrrolidine dithiocarbamate; TRIM, tripartite motif. Fig. S3. Immunofluorescence staining of tight junction proteins ZO-1 and Occludin. Blue: DAPI, Green: ZO-1 or Occludin. Scale bar: 50 μm. a In HRCECs, TRIM46 was interfered with or overexpressed for 24 h. After the cells grew to confluence, the cells were then treated with HG (25 mM). Control osmotic pressure was controlled with normal glucose concentration of 5.5 mM and mannitol. b HRCECs transfected with oeTRIM46 or Vector with overexpression of IκBα (oeIκBα). After the cells grew to confluence, cell permeability analysis was performed (Figure 3d-e). c HRCECs were transfected with oeTRIM46 or Vector. After the cells grew to confluence, cells were administered with the NF-κB inhibitor PDTC (10 μM) and HG [file 40662_2022_305_MOESM1_ESM.docx]

**Additional file 1**

**Immunofluorescence staining**

After washing twice in phosphate-buffered saline (PBS), cells cultured on the coverslips were fixed in 4% paraformaldehyde for 30 min, and then blocked with 5% BSA at room temperature for 1 h. The cells were incubated with rabbit anti-ZO-1 (Abcam, ab221547) or anti-Occludin (Abcam, ab216327) overnight at 4°C. Cells were washed three times with PBS, and then incubated with the [Alexa Fluor 488-labeled goat anti-rabbit IgG(H+L)](http://www.beyotime.com/product/A0453.htm) (Beyotime Biotech.) at room temperature for 1 h. After washing three times with PBS, 4'-6-diamidino-2-phenylindole (DAPI, Beyotime Biotech.) was used to stain nuclei.

**Figure S1**  TRIM46 overexpression and short hairpin RNA (shRNA)-mediated interference of TRIM46 in HRCECs. (a) Protein expression of TRIM46 in HRCECs transfected with oeTRIM46 or Vector. (b) Protein expression of TRIM46 in HRCECs transduced with TRIM46 shRNAs (shTRIM46-1, shTRIM46-2 and shTRIM46-3) or control shRNA (shNC). HRCECs, human retinal capillary endothelial cells; TRIM, tripartite motif

**
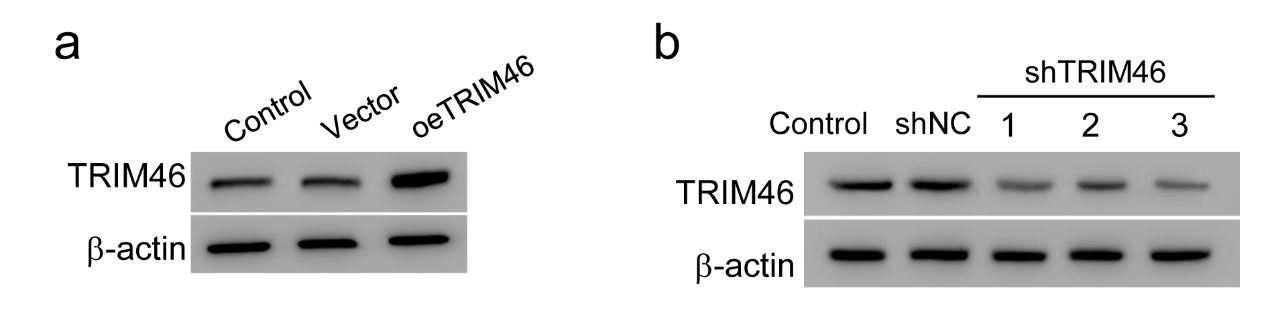
**

**Figure S2.** The images of the monolayers are shown (scale bar: 25 μm). (a) In HRCECs, TRIM46 was interfered with or overexpressed for 24 h. After the cells grew to confluence, the cells were then treated with HG (25 mM). Control osmotic pressure was controlled with normal glucose concentration of 5.5 mM and mannitol. (b) HRCECs transfected with oeTRIM46 or Vector with overexpression of IκBα (oeIκBα). After the cells grew to confluence, cell permeability analysis was performed (Figure 3d-e). (c) HRCECs were transfected with oeTRIM46 or Vector. After the cells grew to confluence, cells were administered with the NF-κB inhibitor PDTC (10 μM) and HG (25 mM). Cell permeability analysis was then performed (Figure 4e-f). HG, high glucose; HRCECs, human retinal capillary endothelial cells; NF-κB, nuclear factor kappa B; PDTC, pyrrolidine dithiocarbamate; TRIM, tripartite motif


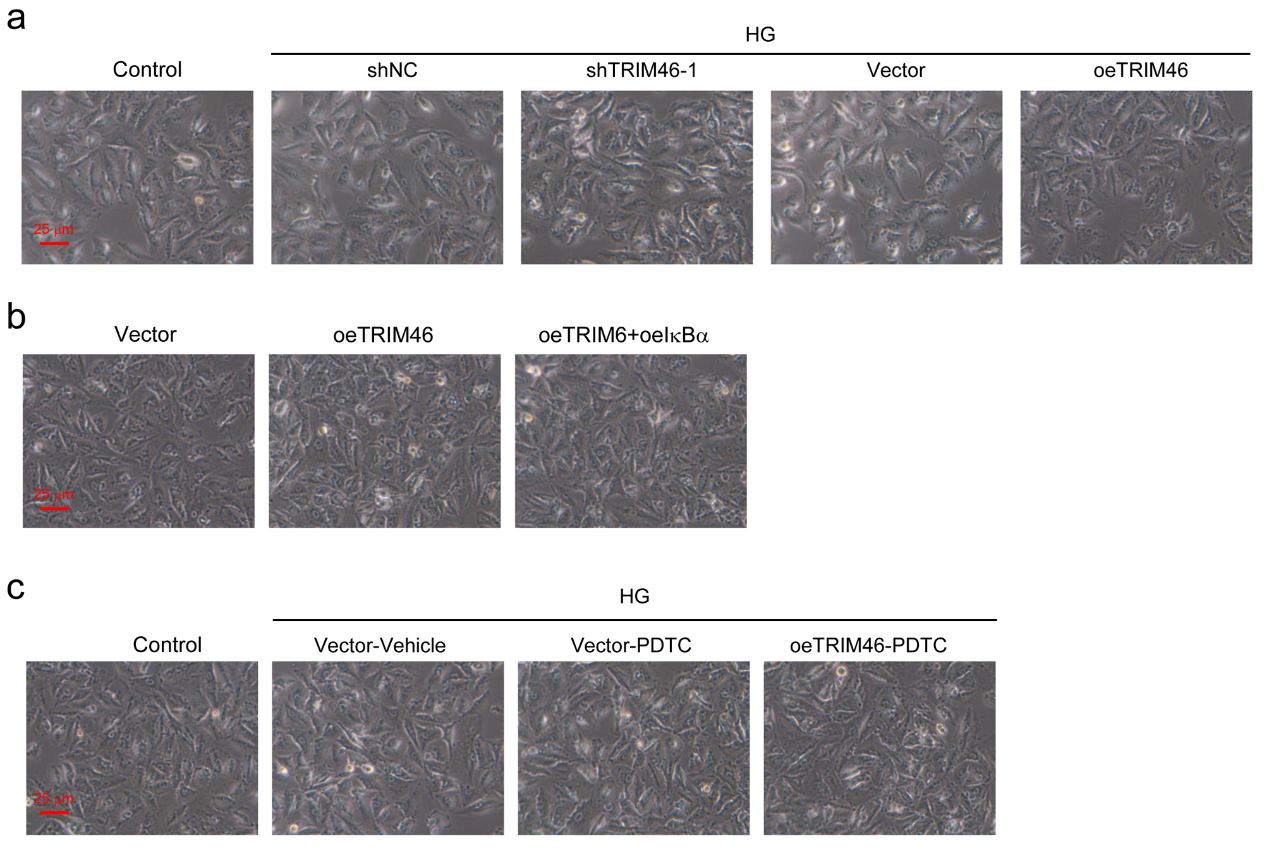


**Figure S3.** Immunofluorescence staining of tight junction proteins ZO-1 and Occludin. Blue: DAPI, Green: ZO-1 or Occludin. Scale bar: 50 μm. (a) In HRCECs, TRIM46 was interfered with or overexpressed for 24 h. After the cells grew to confluence, the cells were then treated with HG (25 mM). Control osmotic pressure was controlled with normal glucose concentration of 5.5 mM and mannitol. (b) HRCECs transfected with oeTRIM46 or Vector with overexpression of IκBα (oeIκBα). After the cells grew to confluence, cell permeability analysis was performed (Figure 3d-e). (c) HRCECs were transfected with oeTRIM46 or Vector. After the cells grew to confluence, cells were administered with the NF-κB inhibitor PDTC (10 μM) and HG (25 mM). Cell permeability analysis was then performed (Figure 4e-f). HG, high glucose; HRCECs, human retinal capillary endothelial cells; NF-κB, nuclear factor kappa B; PDTC, pyrrolidine dithiocarbamate; TRIM, tripartite motif
